# Explaining adoption of AI tools in education: a dual-path model of ethical concern and functional value

**DOI:** 10.3389/fpsyg.2025.1735913

**Published:** 2026-01-21

**Authors:** Tao Yu, Yihuan Tian, Qianghong Huang, Zuling Cheng, Ru Zhang

**Affiliations:** 1 College of Art & Design, Nanning University, Nanning, China; 2 Department of Smart Experience Design, Graduate School of Techno Design, Kookmin University, Seoul, Republic of Korea; 3 Culture Design Lab, Graduate School of Techno Design, Kookmin University, Seoul, Republic of Korea; 4 Department of Global Convergence, Kangwon National University, Chuncheon-si, Republic of Korea

**Keywords:** AIGC, behavioral intention, educational technology, perceived ethical concern, protection motivation theory, technology acceptance model

## Abstract

**Introduction:**

As Artificial Intelligence-Generated Content (AIGC) tools (e.g., ChatGPT for writing assistance, Midjourney for image generation) diffuse into educational settings, their adoption reflects a psychological interplay between functional appraisals and ethical concerns. This study proposes and tests a dual-path model integrating the Technology Acceptance Model (TAM) with Protection Motivation Theory (PMT), incorporating Perceived Ethical Concern (PEC) and Moral Sensitivity (MS).

**Methods:**

Ten latent constructs were modeled: Perceived Severity (PS), Perceived Vulnerability (PV), Self-Efficacy (SE), Response Efficacy (RE), Perceived Ease of Use (PEOU), Perceived Usefulness (PU), PEC, MS, Behavioral Intention (BI), and Continuance Intention (CI). Using structural equation modeling based on data from 589 respondents with prior AIGC experience, we evaluated 14 hypotheses.

**Results:**

The results support the TAM pathway: PU and PEOU positively predict BI and CI. Meanwhile, PMT components operate indirectly; RE and SE influence appraisals by elevating PU and mitigating PEC, whereas PS and PV elevate PEC. PEC shows a significant negative effect on BI and an indirect negative impact on CI. Notably, this negative PEC-BI association is more pronounced among individuals with higher MS.

**Discussion:**

The findings extend psychological accounts of AI tool adoption by jointly modeling moral appraisal and functional value in educational contexts. Furthermore, the study offers actionable implications for platform design and policy, suggesting that improving usability and efficacy cues while increasing ethical transparency can foster responsible, sustained use.

## 1 Introduction

In recent years, with the continuous breakthroughs in Generative Artificial Intelligence technologies and the maturation of large-scale model infrastructures, Artificial Intelligence-Generated Content (AIGC) tools have rapidly permeated the field of education (Zawacki-Richter et al., 2019). Tools such as ChatGPT, Notion AI, Writesonic, and Copilot are widely used for various tasks including text generation, language refinement, question answering, code assistance, and even academic writing (Dwivedi et al., 2021). Their powerful semantic generation capabilities not only significantly enhance teaching and learning efficiency but also break down the high barriers of professional expertise and time investment traditionally associated with content creation (Ocen et al., 2025). In particular, within higher education settings, both students and teachers have integrated AIGC tools into daily educational practices at an unprecedented pace, using them for assignment support, thesis writing, project proposal generation, courseware design, translation and editing, and self-directed learning (Lo, 2023). This phenomenon is part of a broader digital transformation in education, where AI is not merely a tool but a catalyst for reshaping learning ecosystems and skill requirements (Zarifis and Efthymiou, 2022; Naidoo, 2023). As highlighted in recent studies, the integration of AI necessitates a shift from traditional instruction to adaptive, technology-enhanced pedagogical frameworks (Rughiniș et al., 2025). Meanwhile, some educational platforms have also begun actively integrating AIGC-based interface functions to enhance interactivity and personalization in teaching (Smutny and Schreiberova, 2020). These developments also reflect the growing need for sustainable, scalable, and learner-centered educational infrastructures that leverage AI responsibly over the long term.

On one hand, AIGC tools are seen as powerful assistive technologies that can alleviate student workload and improve instructional quality. On the other hand, concerns are mounting over issues such as potential overreliance, unverifiable information authenticity, uncontrollable content generation, and unclear accountability. These issues raise serious questions regarding the legitimacy, rationality, and ethical boundaries of AIGC use in educational contexts (Floridi and Chiriatti, 2020; Cotton et al., 2024). For instance, some educators have reported academic misconduct stemming from students’ use of AIGC tools for writing assignments. Students themselves have noted that reliance on these tools weakens independent thinking. In response to increasing incidents of AIGC-enabled cheating, several universities have even temporarily adjusted their assessment mechanisms (Smutny and Schreiberova, 2020). Although a growing body of research has explored the functional features and application value of AIGC technologies, most studies remain focused on usability and technology adoption pathways, with limited attention to users’ ethical concerns or moral perceptions in decision-making processes (Karran et al., 2024). In real-world educational settings, users often base their decision to adopt AIGC tools not solely on perceived usefulness but on a psychological weighing of potential risks. For example, a student may believe that AIGC significantly improves writing efficiency Perceived Usefulness (PU), yet simultaneously worry that its use could lead to plagiarism Perceived Ethical Concern (PEC). This coexistence of tool efficacy and moral risk renders the adoption decision-making process more complex and fraught with uncertainty (Ravšelj et al., 2025).

While most existing studies have employed the Technology Acceptance Model (TAM) or the Unified Theory of Acceptance and Use of Technology (UTAUT) to explore user behavior, these models primarily focus on technological factors such as PU and Perceived Ease of Use (PEOU) and their effects on Behavioral Intention (BI). However, they fall short in explaining users’ cognitive conflicts and behavioral responses when faced with ethical dilemmas (Ghimire and Edwards, 2024). In contrast, the Protection Motivation Theory (PMT) emphasizes individuals’ cognitive appraisals of threats and coping strategies. It has been widely applied in research on health behavior, security behavior, and AI-related ethical risk, demonstrating strong explanatory power for risk-related cognition (Shrivastava, 2025). Nevertheless, an integrated behavioral model that combines TAM and PMT—while incorporating ethical cognition and individual moral traits—remains lacking, especially for systematically explaining users’ adoption mechanisms and behavioral patterns regarding AIGC tools in educational contexts.

To address this gap, this study constructs a dual-pathway structural model by integrating TAM and PMT, which incorporates both a “functional cognition pathway” and an “ethical cognition pathway.” The model introduces PEC as a key ethical cognition variable and Moral Sensitivity (MS) as a moderating variable, aiming to investigate users’ cognitive mechanisms, psychological trade-offs, and BI when engaging with AIGC tools in educational settings (Chenoweth et al., 2009; Yang, 2024). Specifically, this study seeks to achieve the following three objectives:

1. To examine how threat appraisal and coping cognition influence users’ perceptions of the usefulness of AIGC tools and their recognition of ethical issues;
2. To analyze how both the technological acceptance pathway and the ethical risk pathway jointly affect users’ BI;
3. To investigate how MS moderates the impact of ethical cognition on user behavior.

Through these objectives, this study aims to contribute to theory by addressing the lack of ethical considerations in existing technology adoption models, and to practice by offering ethically informed strategies for educational platforms and policy makers seeking to promote sustainable and inclusive adoption of AI technologies in education.

## 2 Literature review

### 2.1 Research progress on AIGC tools in the field of education

From the functional perspective, numerous studies have demonstrated that AIGC tools hold significant potential as learning aids. Chen found that students using AIGC tools during writing exercises significantly improved their structural expression and task completion efficiency (Chen et al., 2024). Lukas noted that AIGC systems provide learners with real-time feedback, rewriting suggestions, and translation support, thereby alleviating language barriers and cognitive burdens (Jürgensmeier and Skiera, 2024). Crompton and Burke further emphasized that AIGC technologies enhance classroom interaction and promote personalized instruction, showing high adaptability and scalability, particularly in higher education and open learning environments (Crompton and Burke, 2024). Across these studies, PU and PEOU have consistently been validated as key cognitive factors influencing students’ BI, thus reinforcing the core theoretical logic of TAM.

However, as AIGC tools become increasingly embedded in critical teaching activities—such as course writing, academic translation, and assessment completion—associated ethical concerns have become more prominent. First, the risk of academic misconduct has significantly increased. Existing research indicates that students often struggle to distinguish between original and AI-generated content, leading to frequent incidents of plagiarism and academic dishonesty (Plata et al., 2023). Second, AIGC may diminish students’ critical thinking abilities. Zhai (2022) argued that overreliance on tool-generated content can lead to a “cognitive outsourcing” effect, impeding the development of independent cognitive construction and expressive capabilities. In addition, concerns related to unclear content authenticity, algorithmic opacity, and ambiguous accountability have emerged as critical ethical risks for both educators and platform administrators (Ateeq et al., 2024). For instance, Yakubu et al. (2025), in a UTAUT-based study on college students’ adoption of AIGC writing tools, included only “social influence” and “facilitating conditions” as key predictors, without considering negative cognitive variables such as ethical concern. From an ethical perspective, several studies have attempted to introduce MS as a key moderating variable in user behavior models, to capture the intensity of individuals’ psychological responses to ethical issues. For instance, Vance et al. (2012) found that MS significantly moderated users’ BI when facing information leakage risks in the context of information security behavior. Liu et al. (2021), in a study on AI algorithm transparency, noted that individuals with high MS are more inclined to respond ethically to algorithmic bias. A summary of representative literature on AIGC applications in education is provided in Table 1.

**Table 1:** Some representative literature on AIGC applications in education.

Aspect	Article	Description
Writing assistance	Imran and Almusharraf (2023)	ChatGPT reshapes academic writing, requiring new training and safeguards for integrity.
	Mondal and Mondal (2023)	ChatGPT aids academic writing but raises concerns over reliability and ethical risks.
	Mahapatra (2024)	Findings show ChatGPT positively enhances students’ academic writing, with strong student approval.
Learning Efficiency	Guo et al. (2024)	AIGC enhances student motivation through personalized and interactive learning, while raising new challenges for educational practice and ethics.
	Chen et al. (2024)	AIGC is transforming education, led by the US, with research centered on assessment, instructional use, improved learning, and future directions.
	Dai (2024)	AIGC boosts design education by enhancing ideation, prototyping, and personalization, though integration and ethics require further research.
Instructional design	Yilmaz and Karaoglan Yilmaz (2023)	AI-augmented teaching enhances programming skills and educational outcomes by integrating advanced teaching methods with AI models.
	Li (2024)	AIGC technology enhances blended education in finance and economics by boosting effectiveness and fostering creative thought processes, but requires training teachers and ensuring data security.
	Huang and Wu (2024)	AIGC transforms television by automating scripts, virtual hosts, and editing, boosting quality, efficiency, and viewer engagement.
Adoption intention	Lu et al. (2024)	This study applies TAM and TPB to examine teachers’ intention to use generative AI, finding that perceived usefulness and subjective norms significantly influence adoption.
	Ravšelj et al. (2025)	This global study found that most higher education students responded positively to ChatGPT, considering it useful for learning and research.
Ethical risk	Nguyen et al. (2023)	This paper examines AI ethics in education, reviews existing policies, and emphasizes the need for a clear ethical framework.
	Susnjak and McIntosh (2024)	This study assesses ChatGPT’s potential misuse in online exams, emphasizing risks to academic fairness and integrity.
	Ardito (2025)	This paper critiques the effectiveness and ethics of AI detection tools in higher education, calling for a reevaluation of academic integrity practices.
Psychological cognition	Jose et al. (2025)	This study examines the cognitive paradox of AI in education, balancing learning enhancement with cognitive offloading, and highlights the need for responsible use.
	Azeem and Abbas (2025)	This study explores how personality traits influence generative AI use in academia, with perceived fairness moderating the relationship.
	Abbas et al. (2024)	This study analyzes the reasons and consequences of college students using generative AI, noting that while it can support learning, it may also lead to dependency and a decline in critical thinking skills.
Academic integrity	Cotton et al. (2024)	This study explores how to ensure academic integrity in the era of ChatGPT, analyzes the impact of AI on academic misconduct, and proposes corresponding countermeasures.
	McIntire et al. (2024)	Students often choose to plagiarize and cheat as a pragmatic mitigation of risk in academic pursuits, rather than an ethical issue.
Policy and regulatory frameworks	Chan (2023)	This paper proposes a comprehensive AI education policy framework covering teaching, governance, and operations, aiming to guide the application of AI in university administration.
	Zhou and Li (2023)	This study explores the impact of generative AI technologies like ChatGPT on the modernization of educational governance and proposes building an education governance system based on “a core with diversified governance.”
	Li et al. (2025)	This study conducts a comparative analysis of policy documents from universities in the United States, Japan, and China, and proposes a University Policy Development Framework for Generative AI Governance (UPDF-GAI).

### 2.2 Theoretical foundations of user adoption behavior: TAM and PMT

To understand AIGC adoption in education, this study integrates TAM and PMT—two key models explaining how users balance functional benefits with perceived ethical risks in decision-making. These theoretical perspectives are particularly valuable for understanding responsible technology adoption in sustainable educational environments. Originally proposed by Davis (1989), TAM is one of the most widely used frameworks in the information systems domain for predicting user behavior. It posits that two cognitive evaluations—PU and PEOU—are the key determinants of BI to use a new technology (Davis, 1989). PU refers to the belief that using the technology will improve one’s task performance, while PEOU reflects the perceived simplicity of the technology’s operation. Together, these perceptions shape users’ positive evaluations and jointly influence their adoption decisions. Owing to its parsimonious structure and strong predictive validity, TAM has been widely applied in diverse fields such as educational technology, healthcare systems, and e-government (Scherer et al., 2019; ElKheshin and Saleeb, 2020; Wang et al., 2022). In studies related to AIGC, scholars have confirmed the significant impact of PU and PEOU on user adoption. For example, Li (2023) identified PU as one of the strongest predictors of adoption intention among university students using AI-based writing tools; moreover, PEOU was found to positively influence PU, thereby validating the chained causal structure within the TAM framework.

Despite its emphasis on technological performance evaluations in shaping adoption decisions, TAM pays insufficient attention to users’ psychological defense mechanisms and ethical evaluation processes when facing emerging technologies (Islam et al., 2014). This limitation becomes particularly salient in the context of AIGC, which involves complex issues such as moral judgment, responsibility attribution, and value conflicts (Ajibade, 2018). To address this theoretical gap, this study introduces PMT as the foundation for the risk perception pathway. Initially developed by Rogers to explain individuals’ protective behaviors in response to health threats (Floyd et al., 2000), PMT has since been widely applied to areas such as information security (Sommestad et al., 2015), data privacy (Boerman et al., 2021), and AI technology adoption (Park et al., 2024). The core of PMT involves two cognitive stages—threat appraisal and coping appraisal—through which individuals assess risks and decide whether to adopt protective behaviors. In education, these appraisals influence whether AIGC tools are seen as supporting sustainable and equitable learning. Threat appraisal includes Perceived Severity (PS) and Perceived Vulnerability (PV), while coping appraisal comprises Self-Efficacy (SE) and Response Efficacy (RE), reflecting users’ confidence in handling risks and the perceived effectiveness of coping strategies.

Previous research has demonstrated the predictive validity of PMT in explaining user avoidance, resistance or behavioral adjustment in response to technological risks. For example, Park et al. (2024) found that in AI-based virtual service contexts, both PS and PV significantly influenced users’ adoption attitudes and BI, while SE and RE enhanced users’ perceptions of strategic effectiveness and increased their willingness to adopt such technologies. In the cybersecurity domain, Dodge et al. (2023) also identified SE and RE as key predictors of users’ adoption of recommended protective behaviors. Moreover, Su et al. (2022), in the tourism industry context, noted that PS and PV not only shaped practitioners’ risk awareness but also affected their SE and RE, which in turn strengthened their professional resilience and behavioral adjustment capabilities. Integrating PMT with TAM helps construct a comprehensive behavioral model that encompasses both positive motivations (functional adoption pathway) and negative motivations (risk defense pathway). In the context of educational applications of AIGC tools, users may simultaneously hold high functional expectations and ethical or risk-related concerns. Relying solely on TAM is insufficient to fully capture the internal psychological conflicts and dynamic trade-offs users experience (Hsiao and Tang, 2024). More importantly, the four cognitive antecedents in PMT may not only directly influence users’ risk perceptions but also indirectly affect their evaluations of the tool’s functionality, thereby impacting their BI (Hsu and Silalahi, 2024). This potential chain mechanism—from risk perception to functional evaluation to behavioral response—offers a theoretically grounded and practically relevant framework that surpasses TAM in explaining actual user behavior, especially when applied to sustainable technology adoption strategies in education. Accordingly, this study builds upon the primary pathway of TAM by further incorporating the four cognitive antecedents of PMT as predictors of PU and PEC. This dual-pathway adoption behavior model not only extends the explanatory scope of traditional technology acceptance theories but also provides a more systematic theoretical foundation for understanding user behavior in ethically sensitive educational settings involving AIGC tools (Hsu and Silalahi, 2024).

### 2.3 The role of ethical cognition and moral psychology in technology adoption

In the field of technology acceptance and user behavior research, ethical dimensions have long been situated at the periphery. Particularly in the context of educational artificial intelligence tools, users’ concerns over potential moral risks, value conflicts, and normative uncertainties are often simplified as “cognitive burdens” or “usage barriers.” However, as ethical issues surrounding artificial intelligence technologies gain increasing attention from both society and academia, a growing number of scholars have begun to focus on the mechanisms of ethical cognition during technology adoption, attempting to incorporate “ethical judgment” into behavioral intention prediction models (Kwon et al., 2020). Among these concepts, PEC—a risk-oriented ethical cognition—refers to an individual’s subjective perception of moral conflict, ambiguous responsibility, system manipulativeness, and potential adverse consequences during technology use. Initially prominent in research on data privacy, algorithmic fairness, and AI transparency, PEC has since been introduced into studies of user acceptance of AI systems. In the context of educational AI, Sain and Lawal (2024) found that students’ recognition of ethical risks associated with content generation tools significantly and negatively predicted their usage intentions, with heightened sensitivity observed in high-risk scenarios such as academic writing and course assessments. Specific manifestations of PEC in AIGC applications include lack of content originality, unclear attribution of responsibility, model bias risks, increased student dependency, and unbalanced evaluation mechanisms by instructors. Hsiao and Tang (2024) argued that the challenges posed by AIGC in educational settings are not merely technical but constitute a fundamental disruption to the legitimacy of knowledge production. Thus, conceptualizing PEC as a key cognitive factor influencing BI not only responds to the ethical realities introduced by AI technologies but also enhances the model’s capacity to capture complex psychological structures. Moreover, understanding ethical cognition is essential for ensuring the responsible integration of AIGC tools into education systems that aim to be sustainable and socially accountable.

Notably, the effect of ethical cognition on BI is not uniform across all user groups. Individuals may respond differently to the same ethical issue, and this variation often stems from differences in moral psychological traits. One such trait is MS—an individual’s ability to recognize and respond to moral cues in ethically charged situations—which serves as a critical moderating variable in the relationship between PEC and BI (Crowell et al., 2008). According to the Four-Component Model (Rest et al., 1999), MS constitutes the initial stage of activating moral judgment, moral motivation, and moral action; without the recognition of a moral issue in a given context, subsequent judgment processes are unlikely to be triggered. In user behavior studies, MS is often treated as a moderator that explains variations in behavioral responses to ethical cognition. Mower (2018) revealed that individuals with high MS are more inclined to adopt rejection or avoidance strategies when facing moral dilemmas Although originally applied in domains such as organizational behavior and medical ethics, MS—as a psychological trait at the individual level—has proven to be theoretically adaptable and empirically valid in AI ethics scenarios. This is particularly relevant in educational contexts, where both students and teachers may experience value conflicts triggered by the use of AIGC tools, and their levels of MS can significantly influence whether PEC translates into actual resistance to usage (Ashford, 2021).

## 3 Research model and methodology

### 3.1 Theoretical integration and model development

#### 3.1.1 Theoretical foundations of the research model: TAM and PMT

Given that AIGC technologies enhance learning efficiency while simultaneously raising various ethical concerns, users’ adoption behaviors are influenced not only by their perceptions of tool effectiveness but also by factors such as perceived moral risks. Therefore, based on established theoretical foundations, this study integrates TAM and PMT to construct a more comprehensive and explanatory user adoption model, incorporating both the functional evaluation pathway and the risk defense pathway (Nikolic et al., 2024). TAM and PMT represent two distinct yet complementary cognitive pathways—function-driven and risk-driven, respectively. In the context of AIGC applications in education, where technological functionality intersects with ethical sensitivity, the integration of these two theories allows for a more holistic understanding of user adoption mechanisms and extends the theoretical scope of TAM. Accordingly, this study proposes a dual-pathway user behavior model and introduces two additional variables—PEC and MS—to enhance the model’s explanatory power under ethically sensitive conditions (Rhim et al., 2021).

#### 3.1.2 Dual-pathway model construction and theoretical framework

In the functional cognition pathway, the model follows the classical structure of TAM, incorporating PEOU and PU as its core variables. PEOU not only positively influences PU but also, together with PU, positively predicts BI, thereby forming the main pathway logic of “ease of use—functional benefit—adoption intention” (Adjekum et al., 2024). In addition, PU exerts an indirect positive influence on CI, reflecting the extended effect of functional cognition on sustained usage behavior. In the ethical cognition pathway, the model introduces PEC as a key mediating variable to capture users’ subjective judgment regarding potential ethical risks associated with AIGC tools, such as moral conflict, ambiguous academic responsibility, and increased tool dependency. PEC negatively predicts BI, embodying the mechanism of “ethical concern—behavioral inhibition” (Sain and Lawal, 2024). To reveal the formation mechanism of PEC, the model incorporates four antecedent variables from PMT: PS and PV positively influence PEC, representing users’ threat appraisal of risk, while SE and RE negatively influence PEC, reflecting the regulatory function of individuals’ coping abilities (Su et al., 2022). Furthermore, these four PMT variables may also indirectly affect PU, indicating that risk cognition may interfere with functional evaluation and thereby indirectly influence BI (Rhim et al., 2021). To account for individual differences in ethical reactions, the model incorporates MS as a moderating variable to examine its effect on the relationship between PEC and BI. Specifically, individuals with high MS are more likely to experience ethical anxiety when faced with the same ethical scenarios, exhibiting a stronger tendency toward behavioral inhibition. In other words, MS amplifies the negative effect of PEC on BI (Ashford, 2021).

### 3.2 Hypotheses development

Based on the proposed structural model and theoretical logic, this section presents a series of research hypotheses focused on the causal relationships among the core variables, thereby providing a theoretical foundation for the subsequent empirical analysis.

#### 3.2.1 Effects of PMT variables on PU

PU, a core construct within TAM, refers to users’ cognitive judgment that AIGC tools can improve their performance or efficiency in educational settings. However, this functional judgment is not isolated from risk assessment. Theoretically, the direct influence of PMT constructs on PU can be explained by the “cognitive verification cost” mechanism. When users perceive high severity or vulnerability (e.g., hallucinations or ethical pitfalls), they are compelled to allocate additional cognitive resources to verify and correct the AI output. This added effort diminishes the net efficiency gain, thereby directly reducing the tool’s perceived usefulness. According to PMT, users’ evaluation of whether a tool is useful is often influenced by their subjective perceptions of potential threats and their confidence in coping with them. First, when users perceive the use of AIGC tools as potentially resulting in serious negative consequences—such as diminished critical thinking or the erosion of students’ originality. Specifically, when their PS is high, their positive evaluation of the tool’s effectiveness may be suppressed, thereby reducing PU (González-Ponce et al., 2024).

*H1*: *PS has a significant negative effect on PU.*

Second, users believe they are personally vulnerable to such negative outcomes (i.e., high PV), they may develop doubts about the tool’s reliability and long-term value, leading to a decline in PU. Empirical studies have shown that higher levels of PV often result in defensive attitudes, which in turn inhibit positive evaluations of technology (Jelovčan et al., 2021).

*H2: PV has a significant negative effect on PU.*

Conversely, when users have strong confidence in their own abilities (i.e., high SE) and believe they can use AIGC tools correctly and safely, they are more likely to focus on the tools’ benefits, which facilitates a higher level of PU. SE has been identified as one of the PMT variables most strongly associated with adoption intention and high performance-related cognition in multiple studies (Hedayati et al., 2023).

*H3: SE has a significant positive effect on PU.*

Moreover, if users believe that proper management systems and guidance protocols are in place to effectively mitigate potential risks (i.e., high RE), their trust in the tool and perceived utility will likely increase. RE can effectively alleviate concerns about risk and shift user attention toward functional benefits (Courneya and Hellsten, 2001).

*H4: RE has a significant positive effect on PU.*

#### 3.2.2 Effects of PMT variables on PEC

PEC reflects users’ subjective attention to the potential moral dilemmas that AIGC tools may provoke in educational settings, such as plagiarism, diminished student accountability, and blurred authorship. According to PMT, users’ subjective assessments of threat severity during the threat appraisal phase significantly influence their level of moral alertness. When users perceive the potential consequences of AIGC tools to be highly destructive (i.e., high PS) or believe they are personally more susceptible to ethical misuse (i.e., high PV), they tend to exhibit stronger moral vigilance, thereby intensifying their PEC (Ruan et al., 2020; Jannat et al., 2024).

*H5: PS has a significant positive effect on PEC.*

*H6: PV has a significant positive effect on PEC.*

Conversely, if users believe they are capable of identifying risks and using the technology responsibly (i.e., high SE) or trust that institutional policies and guidelines can effectively regulate misuse (i.e., high RE), their ethical concerns may be partially mitigated, thus reducing the level of PEC (Block and Keller, 1998; Sher et al., 2017; Al-Sharafi et al., 2021).

*H7: SE has a significant negative effect on PEC.*

*H8: RE has a significant negative effect on PEC.*

#### 3.2.3 TAM pathway: effects of PEOU and PU on BI

According to TAM, users evaluate technology primarily based on PEOU and PU. Numerous empirical studies have shown that PEOU not only reduces cognitive and operational costs by simplifying the usage process but also significantly enhances users’ overall perception of a tool’s utility, thereby influencing their intention to adopt it. Specifically, when users perceive a system as easy to operate, their evaluation of its value tends to improve, leading to a heightened level of PU (Rahman, 2018).

*H9: PEOU has a significant positive effect on PU.*

Furthermore, PEOU can directly reduce usage barriers and psychological resistance, thereby enhancing BI. Prior research suggests that users’ perceptions of intuitive usability can boost their confidence and willingness to adopt a system, especially during initial encounters with the technology (Khosrow-Pour, 2003).

*H10: PEOU has a significant positive effect on BI.*

PU, a core construct of TAM, reflects users’ expectations regarding the benefits of using a technology. The positive relationship between PU and BI has been repeatedly validated in studies on AI technology adoption. The more users believe a given tool can improve their learning or work efficiency, the more likely they are to adopt it (Xu et al., 2022; Ali et al., 2024).

*H11: PU has a significant positive effect on BI.*

#### 3.2.4 Ethical pathway: the effect of PEC on BI

While PU and PEOU can stimulate users’ intention to adopt a technology, in ethically sensitive contexts, the risk-related concerns evoked by PEC may counteract the positive effects of functional cognition. When users believe that the use of AIGC tools may violate educational fairness, undermine student autonomy, or generate issues related to academic responsibility, they may consciously avoid adopting the tool—even if its functional benefits are evident (Chiu and Kuo, 2007).

*H12: PEC has a significant negative effect on BI.*

#### 3.2.5 Continuance pathway and the moderating role of MS

BI serves as a key antecedent to CI. Once users develop a clear intention to adopt a tool, they typically move toward forming habitual usage patterns, reflected in their continued use of the technology (Jeong et al., 2025). Empirical research across various digital platforms and AI application contexts has consistently confirmed the positive predictive relationship between BI and CI (Zhou et al., 2018; Liu et al., 2023).

*H13: BI has a significant positive effect on CI.*

In addition, users’ responses to ethical information vary significantly. MS, as a psychological trait, describes an individual’s capacity to recognize value conflicts in morally salient situations. When MS is high, users are more likely to perceive the severity of the issues represented by PEC, thereby amplifying the negative effect of PEC on BI. Research in moral education has demonstrated that individuals with high MS are more inclined to translate perceived moral conflicts into avoidance behavior (Strahovnik, 2018).

*H14*: *MS positively moderates the relationship between PEC and BI; that is, the higher the MS, the stronger the negative effect of PEC on BI.*

#### 3.2.6 Proposed research model

**Figure 1:**
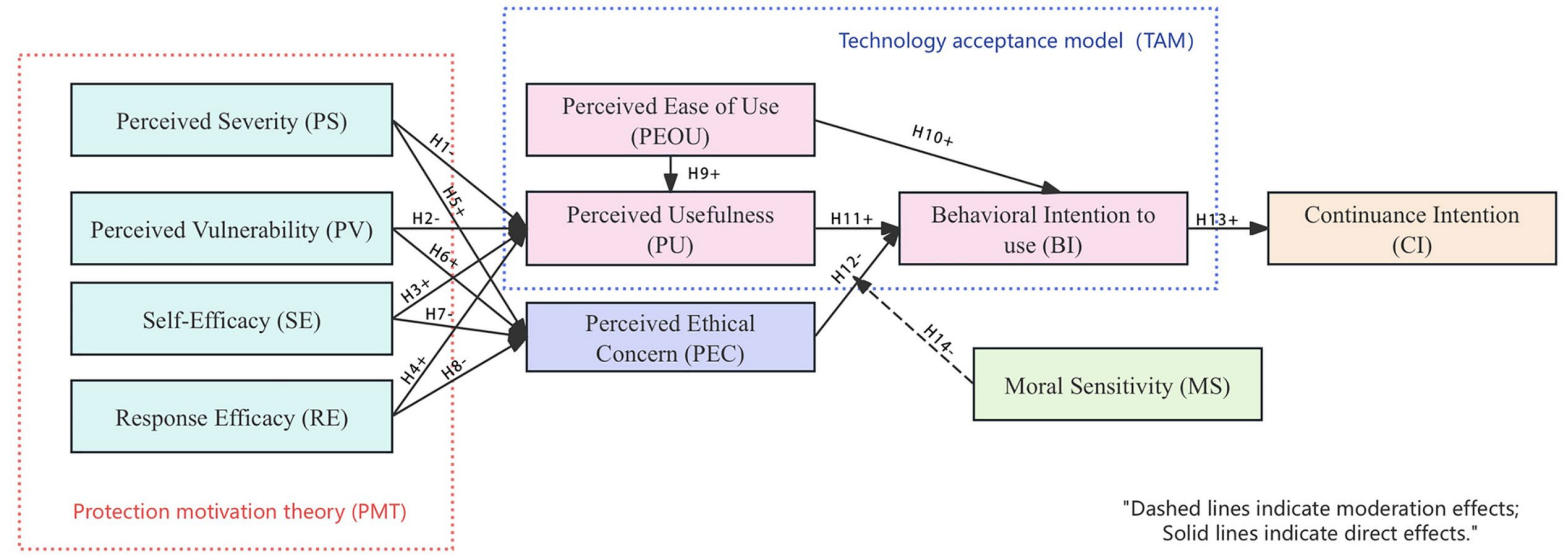
Proposed research model and hypotheses.

In summary, based on the step-by-step development of the hypotheses above, this study constructs an integrated dual-pathway theoretical model that comprehensively considers both functional and ethical factors influencing the adoption of AIGC tools in educational contexts, as illustrated in Figure 1. This model will be empirically tested using Structural Equation Modeling (SEM) in subsequent analyses to evaluate its theoretical validity and explanatory power.

### 3.3 Empirical design and implementation strategy

#### 3.3.1 Variable operationalization and instrument development

A structured questionnaire was developed to measure ten latent variables (i.e., PS, PV, SE, RE, PEOU, PU, PEC, MS, BI, CI), with items adapted from validated scales and refined for the educational AIGC context. All items were measured using a five-point Likert scale. The questionnaire included four sections: study introduction, demographic questions, measurement items (randomized to reduce bias), and closing remarks. Expert reviews and a pilot test (*n* = 30) confirmed the clarity, validity, and contextual suitability of the instrument (see Table 2).

**Table 2:** Questionnaire items.

Variables	Items	Issue	Reference
PS	PS1	I believe that the misuse of AIGC tools in education could lead to serious consequences.	Nguyen and Tang (2022)
	PS2	If AIGC tools generate incorrect information, the impact could be highly detrimental.	
	PS3	I am concerned that AIGC may negatively affect students’ learning outcomes.	
PV	PV1	I believe I am susceptible to issues related to AIGC.	Ateş and Gündüzalp (2025)
	PV2	I may encounter risks or misinformation when using AIGC.	
	PV3	I feel vulnerable to the negative impacts brought by AIGC.	
SE	SE1	I am capable of effectively using AIGC tools to complete learning tasks.	Morales-García et al. (2024)
	SE2	I can identify and avoid potential problems when using AIGC.	
	SE3	I can use AIGC tools effectively even without guidance.	
RE	RE1	By using AIGC properly, I can effectively avoid its potential risks.	Courneya and Hellsten (2001)
	RE2	I believe AIGC can be beneficial for learning if used correctly.	
	RE3	I think appropriate measures can effectively prevent the negative effects of AIGC.	
PEOU	PEOU1	I find AIGC tools easy to use.	Hess et al. (2014)
	PEOU2	It is easy to complete tasks using AIGC tools.	
	PEOU3	Learning how to use AIGC is easy for me.	
PU	PU1	Using AIGC can improve my learning efficiency.	Sukirman et al. (2024)
	PU2	AIGC tools help me accomplish complex tasks.	
	PU3	I believe AIGC is useful in learning.	
	PU4	AIGC can help me achieve better learning outcomes.	
PEC	PEC1	I am concerned that AIGC tools may lead to academic misconduct in education.	Jannat et al. (2024)
	PEC2	Content generated by AIGC may pose problems related to authenticity or ethics.	
	PEC3	I believe relying on AIGC may impair students’ ability for independent learning.	
	PEC4	I think using AIGC in education involves ethical risks.	
MS	MS1	I am sensitive to possible moral issues in the use of technological tools.	Reynolds (2008)
	MS2	I usually think carefully when faced with ethically charged situations.	
	MS3	I pay particular attention to whether technology is being used for legitimate purposes.	
	MS4	I tend to prioritize moral standards in my behavioral decisions.	
BI	BI1	I am willing to use AIGC tools in my future learning.	Ruan et al. (2020)
	BI2	If possible, I intend to frequently use AIGC for learning support.	
	BI3	I plan to continuously explore the use of AIGC in education.	
	BI4	I prefer educational platforms that include AIGC features.	
CI	CI1	I intend to use AIGC tools as long-term learning aids.	Zhou et al. (2018)
	CI2	I am willing to continue using AIGC even if alternative tools are available.	
	CI3	I maintain a positive attitude toward the continued use of AIGC in the future.	

#### 3.3.2 Sampling strategy and survey implementation

This study used non-probability convenience sampling to recruit university students, teachers, and education professionals with prior AIGC experience. A screening question ensured eligibility. A power analysis was conducted using G*Power 3.1 to determine the minimum required sample size. To detect a medium effect size (f^2^ = 0.15) with a statistical power of 0.95 and a significance level of 0.05 (considering 10 predictors in the regression model), the calculated minimum sample size was 172. Our final sample of 589 participants significantly exceeds this requirement, ensuring adequate statistical power for data analysis. A pilot test (*n* = 30) refined item clarity and optimized completion time. The final questionnaire was hosted on Wenjuanxing and distributed through targeted academic networks to ensure relevance. Specifically, recruitment links were shared in university student course groups, faculty professional exchange groups, and educational technology forums on WeChat and QQ. Responses were collected anonymously and voluntarily. Before accessing the questionnaire, all participants were presented with the study’s purpose and privacy policy, and they provided digital informed consent by clicking an ‘I Agree’ button. Data quality was ensured through logical checks, mandatory items, and reverse-coded questions. After data collection, responses were cleaned and prepared in Excel and SPSS for analysis. The survey complied with ethical standards, ensuring anonymity and academic-only use of data.

#### 3.3.3 Data analysis procedures and modeling approach

This study adopted SEM using SPSS and AMOS to assess measurement quality and test hypotheses. The process included: (1) data screening (checking for missing values and outliers) and descriptive statistics; (2) reliability and validity testing via Cronbach’s *α*, Composite Reliability (CR), and Average Variance Extracted (AVE); (3) Confirmatory Factor Analysis (CFA) to assess model fit using indices such as CMIN/DF, CFI, TLI, RMSEA, and SRMR. After validating the measurement model, structural path analysis was conducted. The moderating effect of MS was tested through multi-group SEM and interaction-term regression. Discriminant validity was confirmed by comparing AVE with squared inter-construct correlations. Model robustness was ensured via multiple fit indices to avoid overfitting and confirm theoretical coherence.

## 4 Results

A total of 620 questionnaires were distributed, with 589 valid responses retained after screening, resulting in a 95.0% effective response rate. The sample showed a near-equal gender distribution (52% male, 48% female), with most respondents aged 19–35 (64%). A combined 72% held bachelor’s or master’s degrees, and 25% held doctoral degrees. Students comprised 61% of the sample, while teachers accounted for 21%. All participants had prior AIGC experience, primarily for translation and editing (28%) and writing support (26%) (see Table 3).

**Table 3:** Demographic characteristics of respondents.

Category	Option	Frequency (n)	Percentage (%)
Gender	Male	308	52.29%
	Female	281	47.71%
Age	18 or below	7	1.19%
	19–25	195	33.11%
	26–35	185	31.41%
	36–45	127	21.56%
	46 or above	75	12.73%
Educational background	High school or below	18	3.06%
	Bachelor’s degree	230	39.05%
	Master’s degree	194	32.94%
	Doctorate or above	147	24.96%
Occupation	Undergraduate	206	34.97%
	Graduate student	155	26.32%
	Teacher	121	20.54%
	Employee of education platform	80	13.58%
	Other	27	4.58%
AIGC tool usage frequency	Occasionally	314	53.31%
	Frequently	275	46.69%
Purpose of AIGC tool usage	Writing assistance	152	25.81%
	Translation and editing	164	27.84%
	Academic Q&A	128	21.73%
	Courseware development	103	17.49%
	Other	42	7.13%

Reliability analysis was conducted to evaluate the internal consistency of questionnaire items, indicating how well they measure the same construct. As shown in Table 4, all ten constructs in the questionnaire exceeded Cronbach’s alpha values above the 0.70 threshold, confirming satisfactory internal consistency. These results confirm that the measurement scales used in this study are reliable.

**Table 4:** Reliability analysis of questionnaire constructs.

Construct	Number of items	Cronbach’s alpha
PS	3	0.784
PV	3	0.774
SE	3	0.792
RE	3	0.830
PEOU	3	0.813
PU	4	0.877
PEC	4	0.853
MS	4	0.891
CI	3	0.809
BI	4	0.802

**Figure 2:**
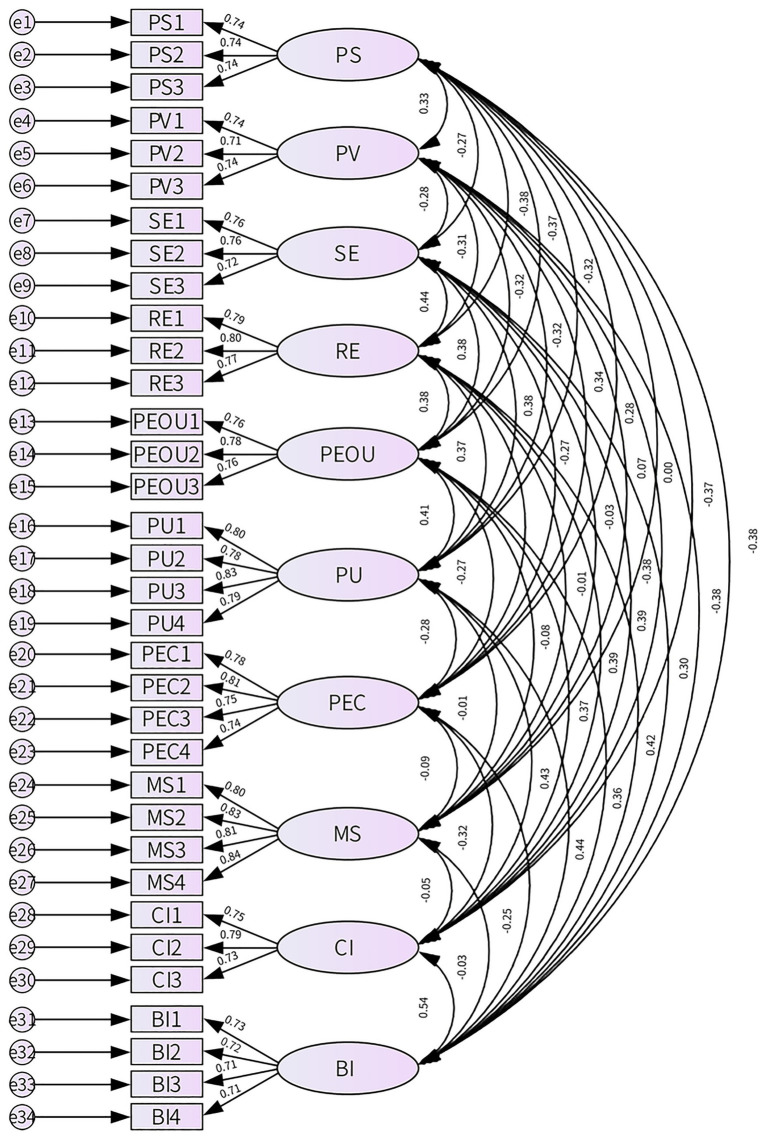
Standardized output confirmatory factor analysis model diagram.

Validity testing in this study examined both content and construct validity. Content validity was ensured by adapting measurement items from established literature and refining them through preliminary analysis. Construct validity was assessed via CFA, with all model fit indices meeting recommended thresholds (e.g., CMIN/DF = 1.086, GFI = 0.951, RMSEA = 0.012, CFI = 0.995), indicating a good model fit (see Table 5 and Figure 2). These results confirm the robustness of the measurement model. The following section examines convergent and discriminant validity.

**Table 5:** Confirmatory factor analysis (CFA) model fit indices.

Fit index	Recommended threshold	Observed value	Evaluation
CMIN/DF	<3	1.086	Excellent
GFI	>0.80	0.951	Excellent
AGFI	>0.80	0.940	Excellent
RMSEA	<0.08	0.012	Excellent
NFI	>0.9	0.942	Excellent
IFI	>0.9	0.995	Excellent
TLI	>0.9	0.994	Excellent
CFI	>0.9	0.995	Excellent
PNFI	>0.5	0.809	Excellent
PCFI	>0.5	0.855	Excellent

Convergent validity was evaluated using AVE and CR. As shown in Table 6, all constructs met the recommended thresholds (AVE > 0.50, CR > 0.70). Furthermore, standardized factor loadings exceeded 0.70 and were statistically significant (*p* < 0.001). These results confirm the strong convergent validity and internal consistency of the measurement model.

**Table 6:** Results of confirmatory factor analysis (CFA).

Construct	Observed variable	Factor loading	S.E.	C.R.	*p*	CR	AVE
PS	PS1	0.740				0.785	0.548
	PS2	0.741	0.065	14.728	***		
	PS3	0.741	0.072	14.734	***		
PV	PV1	0.738				0.775	0.535
	PV2	0.715	0.064	14.119	***		
	PV3	0.740	0.072	14.333	***		
SE	SE1	0.760				0.793	0.561
	SE2	0.761	0.065	15.538	***		
	SE3	0.725	0.067	15.148	***		
RE	RE1	0.793				0.830	0.620
	RE2	0.796	0.055	18.194	***		
	RE3	0.773	0.056	17.844	***		
PEOU	PEOU1	0.761				0.813	0.593
	PEOU2	0.784	0.063	16.745	***		
	PEOU3	0.764	0.065	16.517	***		
PU	PU1	0.804				0.877	0.641
	PU2	0.778	0.049	19.963	***		
	PU3	0.834	0.047	21.624	***		
	PU4	0.786	0.047	20.227	***		
PEC	PEC1	0.776				0.854	0.594
	PEC2	0.812	0.054	19.164	***		
	PEC3	0.755	0.056	17.909	***		
	PEC4	0.737	0.057	17.462	***		
MS	MS1	0.802				0.892	0.673
	MS2	0.828	0.046	21.710	***		
	MS3	0.814	0.046	21.283	***		
	MS4	0.838	0.045	22.019	***		
CI	CI1	0.751				0.803	0.576
	CI2	0.794	0.066	16.628	***		
	CI3	0.731	0.067	15.804	***		
BI	BI1	0.728				0.811	0.518
	BI2	0.723	0.066	15.405	***		
	BI3	0.713	0.075	15.230	***		
	BI4	0.714	0.072	15.252	***		

***Significance at the 0.001 level.

Discriminant validity was confirmed by comparing the square roots of the AVE with inter-construct correlations (Fornell-Larcker criterion). As shown in Table 7, the square roots of the AVE for each construct were greater than their correlations with other constructs, indicating that the measurement model demonstrates good discriminant validity.

**Table 7:** Discriminant validity analysis (Fornell–Larcker criterion).

Constructs	PS	PV	SE	RE	PEOU	PU	PEC	MS	CI	BI
PS	0.741									
PV	0.325	0.731								
SE	−0.274	−0.282	0.749							
RE	−0.363	−0.311	0.436	0.787						
PEOU	−0.372	−0.321	0.383	0.365	0.77					
PU	−0.321	−0.32	0.384	0.373	0.414	0.801				
PEC	0.278	0.343	−0.269	−0.284	−0.273	−0.258	0.771			
MS	0.001	0.074	−0.032	−0.013	−0.085	−0.007	−0.088	0.821		
CI	−0.373	−0.378	0.39	0.386	0.375	0.429	−0.322	−0.054	0.759	
BI	−0.381	−0.382	0.305	0.417	0.363	0.439	−0.251	−0.029	0.542	0.719

**Figure 3:**
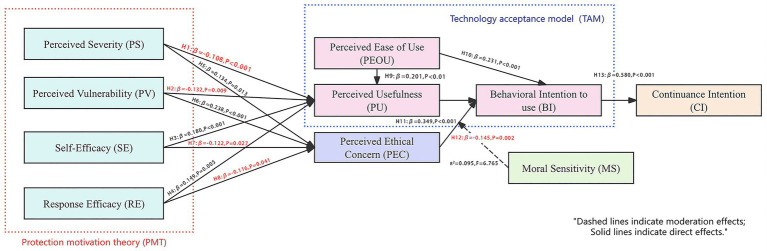
Structural equation model.

This study employed SEM to test the proposed hypotheses. As shown in Figure 3, SEM enabled the analysis of complex causal relationships among latent variables. Model fit was assessed using standard indices to ensure empirical adequacy and theoretical consistency.

As shown in Table 8, all model fit indices met recommended thresholds, indicating a well-fitting structural model. Absolute fit indices (CMIN/DF = 1.297, GFI = 0.948, AGFI = 0.937, RMSEA = 0.022) and incremental fit indices (NFI = 0.935, IFI = 0.984, TLI = 0.982, CFI = 0.984) confirmed a good model fit. Parsimonious indices (PNFI = 0.821, PCFI = 0.864) also exceeded the 0.50 standard. Collectively, these results demonstrate the robust fit of the structural model (see Table 9).

**Table 8:** Model fit indices of the structural equation model (SEM).

Fit index	Recommended threshold	Observed value	Evaluation
CMIN/DF	<3	1.297	Excellent
GFI	>0.80	0.948	Excellent
AGFI	>0.80	0.937	Excellent
RMSEA	<0.08	0.022	Excellent
NFI	>0.9	0.935	Excellent
IFI	>0.9	0.984	Excellent
TLI	>0.9	0.982	Excellent
CFI	>0.9	0.984	Excellent
PNFI	>0.5	0.821	Excellent
PCFI	>0.5	0.864	Excellent

**Table 9:** Path coefficients and hypothesis testing results.

Path	Path coefficient	S.E.	C.R.
PU ← PEOU	0.201***	0.068	3.716
PU ← PS	−0.108*	0.063	−2.066
PU ← PV	−0.132**	0.068	−2.602
PU ← SE	0.180***	0.063	3.336
PU ← RE	0.149**	0.057	2.782
PEC ← PS	0.134*	0.058	2.471
PEC ← PV	0.238***	0.065	4.340
PEC ← SE	−0.122*	0.058	−2.210
PEC ← RE	−0.116*	0.053	−2.044
BI ← EOU	0.231***	0.046	4.375
BI ← PU	0.349***	0.036	6.679
BI ← PEC	−0.145**	0.036	−3.157
CI ← BI	0.580***	0.045	10.407

*p < 0.05, **p < 0.01, ***p < 0.001.

Structural paths were estimated using Maximum Likelihood Estimation (MLE). Critical ratios exceeded ±1.96, indicating that the results were statistically significant (*p* < 0.05). Specifically, PEOU (*β* = 0.201), SE (*β* = 0.180), and RE (*β* = 0.149) positively influenced PU, while PS (*β* = −0.108) and PV (*β* = −0.132) had negative effects. Regarding PEC, PS (*β* = 0.134) and PV (*β* = 0.238) exerted positive effects, whereas SE (*β* = −0.122) and RE (*β* = −0.116) showed negative effects. Furthermore, both PEOU (*β* = 0.231) and PU (*β* = 0.349) positively predicted BI, while PEC (*β* = −0.145) negatively impacted BI. Finally, BI had a strong positive effect on CI (*β* = 0.580). These findings confirm that all hypothesized paths are statistically significant. However, it is important to note that the path coefficients for H1 (PS - > PU, *β* = −0.108) and H8 (RE - > PEC, *β* = −0.116) are relatively weak compared to other structural paths. This suggests that while risk perception factors do influence functional and ethical evaluations, their impact is less dominant than functional drivers like PEOU and PU.

To assess the moderating role of MS, hierarchical regression was conducted using SPSS 26.0. After controlling for demographic variables and AIGC usage patterns, PEC and MS were mean-centered, and an interaction term (PEC × MS) was added. Crucially, as shown in Table 10, the control variable ‘Occupation’ did not show a statistically significant effect on BI (*p* > 0.05). This lack of significant heterogeneity among students, teachers, and platform employees supports the validity of pooling these subgroups for the analysis. Subsequently, the interaction term had a significant negative effect on BI (*β* = −0.121, *p* < 0.001), confirming that MS moderates the PEC–BI relationship. Specifically, higher levels of MS amplify the negative impact of PEC on behavioral intention. The *R*^2^ value for this regression model was 0.095. It should be noted that this value reflects the variance explained specifically by the interaction analysis setup, rather than the full predictive power of the comprehensive structural model. The primary purpose of this regression was to test the significance of the moderating effect of MS, which was confirmed (*p* < 0.001), rather than to maximize variance explanation.

**Table 10:** Moderating effect of moral sensitivity (MS).

Variables	Dependent variable = BI			
	*B*	SE	*t*	*p*
(Constant)	3.731	0.267	13.977	0.000
Gender	−0.120	0.063	−1.917	0.056
Age	0.055	0.030	1.829	0.068
Educational background	0.065	0.037	1.763	0.078
Occupation	−0.022	0.026	−0.866	0.387
Awareness or use of AIGC tools	0.002	0.064	0.035	0.972
Purpose of AIGC tool usage	0.036	0.026	1.404	0.161
PEC	−0.200	0.034	−5.846	0.000
MS	−0.014	0.030	−0.479	0.632
PEC × MS	−0.121	0.027	−4.471	0.000
*R*^2^	0.095			
*F*	6.765			

**Figure 4:**
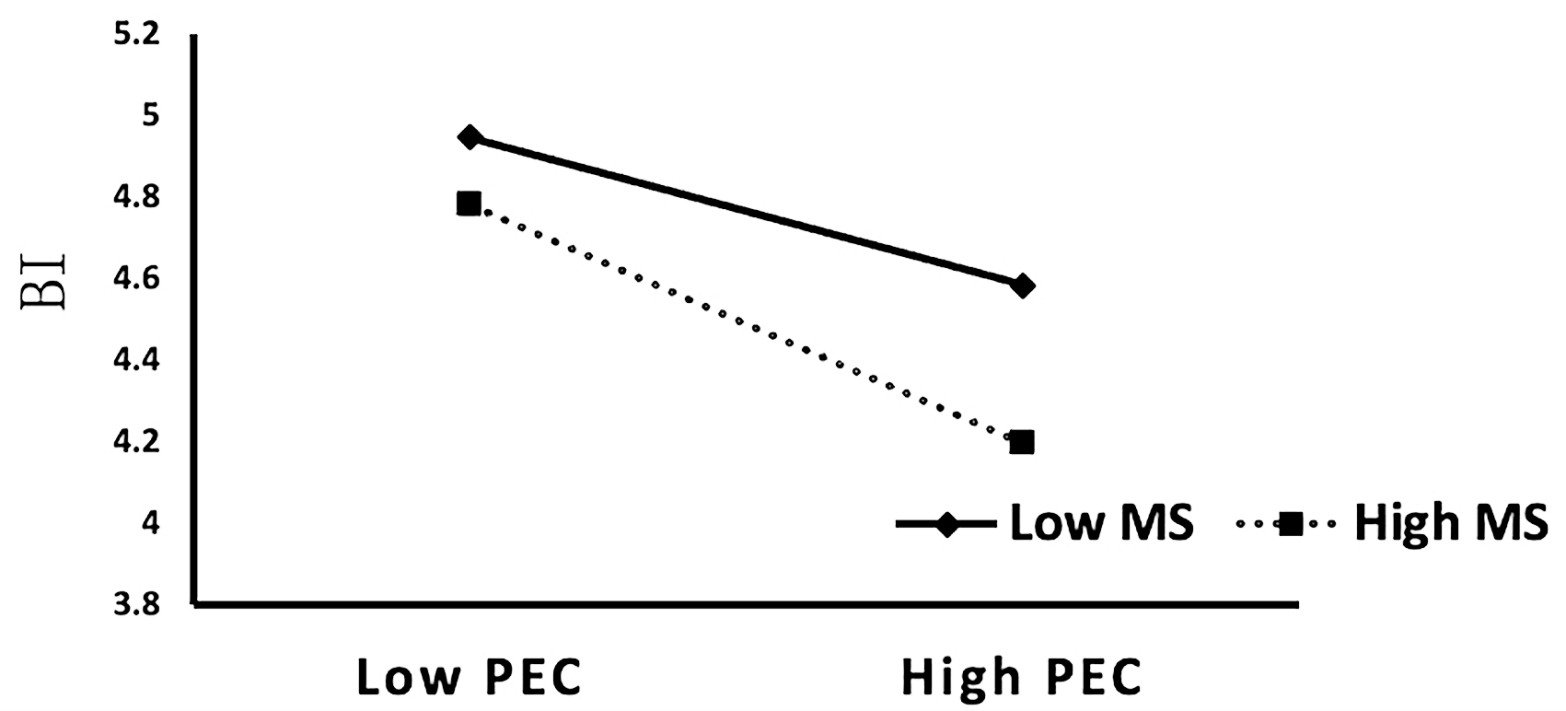
Simple slope analysis of the moderating effect of MS.

Further simple slope analysis (Figure 4) showed that the negative effect of PEC on BI was more pronounced at high levels of MS, and weaker at low levels. This confirms that MS amplifies the inhibitory impact of ethical concerns on behavioral intention, supporting the proposed moderation hypothesis.

## 5 Discussion

### 5.1 Summary of key findings

Building on TAM and PMT, this study developed a dual-pathway adoption model that incorporates two critical variables: PEC and MS. The model systematically investigates users’ adoption mechanisms of AIGC tools within educational settings. Structural equation modeling was used to test 14 hypothesized paths, all of which were found to be statistically significant, indicating a robust model fit and theoretical coherence. In the functional cognition pathway, PEOU had a significant positive effect on both PU and BI (supporting H9 and H10), while PU also significantly enhanced BI and further influenced CI (supporting H13). These findings confirm the applicability of the core TAM framework in the context of AIGC use in education.

In the risk cognition pathway, PS and PV negatively influenced PU but positively affected PEC. Conversely, SE and RE positively influenced PU while negatively affecting PEC. These results support the logic of the PMT framework by showing that both threat and coping appraisals shape users’ perceptions of AIGC tools’ functionality and ethical risks. Within the ethical pathway, PEC significantly and negatively impacted BI, indicating that ethical concerns can suppress users’ willingness to adopt AIGC tools in educational contexts. Moreover, MS significantly moderated the relationship between PEC and BI, with individuals high in MS being more likely to amplify the negative impact of ethical concerns on adoption behavior. Overall, the dual-pathway model successfully integrates functional, risk, and ethical cognitive dimensions, uncovering the multifaceted drivers of user behavior in educational AIGC scenarios. The findings also validate the explanatory power of MS as a key psychological trait, offering both theoretical and methodological contributions to future research on AI in education.

### 5.2 Discussion of functional and risk pathways

Among the positive predictors of PU, PEOU and RE showed the most significant effects. Users who perceive AIGC tools as intuitive, easy to operate, and user-friendly are more likely to positively evaluate their usefulness—this finding aligns with the core logic of TAM as proposed by Davis (1989) (supporting H9). Notably, however, RE also had a strong and statistically significant impact on PU, with an effect size nearly comparable to PEOU (supporting H4). This suggests that in educational contexts, users’ trust in institutional safeguards plays a critical role in their assessment of a tool’s value. In the domain of health technology adoption, prior studies have found that users’ perception of clearly defined rules and governance mechanisms significantly enhances both perceived utility and behavioral intention (Zhang et al., 2017). Similarly, in AI-driven education, higher RE is associated with stronger PU. Particularly in education—a domain characterized by high responsibility—the need for institutional clarity becomes more pronounced, amplifying the role of RE in shaping perceptions (Osman and Yatam, 2024). By contrast, although SE had a statistically significant positive effect on PU (supporting H3), its influence was relatively weaker. This may reflect users’ perception of AIGC tools as “functionally rich but logically complex”—even those with adequate experience may still lack confidence in managing output quality (Masry Herzallah and Makaldy, 2025). This finding echoes Kasneci et al. (2023) argument that although AIGC can enhance efficiency in education, excessive reliance on system logic may reduce users’ perceived control and agency.

Regarding negative influences on PU, the suppressive effects of PS and PV are statistically significant but relatively weak (supporting H1 and H2). This suggests that while users recognize the potential severe consequences of AIGC usage (e.g., hallucinations or bias), these risks do not substantially diminish their perception of the tool’s utility. This ‘utility-over-risk’ calculus implies that in educational settings, the demand for efficiency often outweighs concerns about potential severity. These results support PMT assumption that threat perception weakens positive evaluations (Ruan et al., 2020), and further indicate that users’ risk cognition has deeply permeated their value judgments of AIGC tools. This diverges from the traditional TAM assumption that PU is generally unaffected by negative variables, and can be explained by the contextual specificity of education: AIGC tools are directly linked to critical issues such as student assignments, fair evaluation, and content originality. When users recognize the potential risks—such as encouraging academic laziness or blurring responsibility—they may downgrade their value assessments even if they acknowledge the tool’s efficiency. This aligns with Kelly et al.’s (2023) findings that educational users often hold a dual perception of AIGC as “useful but potentially harmful,” especially in high-stakes scenarios such as examinations and grading, where PU is easily disrupted by ethical evaluations. In addition, the study found that RE had a significant negative effect on PEC. However, this effect was relatively modest (H8: *β* = −0.116). This indicates that simply believing in the effectiveness of external regulations or policy safeguards is not enough to fully eliminate users’ ethical anxieties. Since AIGC ethics involve complex value judgments, institutional responses alone may have a limited capacity to soothe users’ subjective ethical concerns compared to internal factors. RE not only significantly reduces PEC (Al-Sharafi et al., 2021) but also contributes positively to the formation of PU (Zhang et al., 2017). This mechanism can be attributed to RE’s function in building psychological safety: when users believe that there are clear rules and institutional protections, they are more likely to downplay ethical risks and elevate utility evaluations. This finding is consistent (Sain and Lawal, 2024), which shows that in ethically sensitive contexts, trust mechanisms at the organizational or platform level often surpass individual self-efficacy in driving technology acceptance.

Regarding the formation of ethical concerns, the analysis confirmed that threat appraisals (i.e., PS and PV) significantly heightened PEC (supporting H5 and H6). Theoretically, this suggests that risk perception acts as a cognitive trigger for ‘moral vigilance.’ When users believe that AIGC misuse leads to severe consequences (e.g., academic dishonesty) or feel personally susceptible to these risks, their psychological defense mechanisms are activated, manifesting as heightened ethical anxiety. Conversely, coping appraisals (i.e., SE and RE) acted as protective factors, significantly mitigating PEC (supporting H7 and H8). This indicates that a ‘sense of control’ acts as a buffer against ethical distress. When users feel competent in managing the tool SE or trust that external regulations are effective RE, they perceive the ethical risks as manageable rather than overwhelming, thereby lowering their overall level of concern.

Taken together, this study identifies PU as a critical intersection between the TAM and PMT pathways, reflecting a dual dynamic—being suppressed by risk variables while simultaneously promoted by structural trust. This structural tension suggests that in the adoption of educational AI tools, the cognitive weight of PU is shaped not only by perceptions of tool performance but also by users’ subjective assessments of whether the associated risks are manageable. Functional design alone may not be sufficient to drive adoption intentions; rather, psychological assurance must be built through institutional safeguards, user training, and ethical education to establish both a sense of control and trust (Masry Herzallah and Makaldy, 2025).

### 5.3 Ethical mechanisms and moderating effects (PEC and MS)

In this study, PEC emerged as a significant negative predictor of BI. Although its path coefficient (*β* = −0.145) is smaller than that of functional drivers such as PU (*β* = 0.349) and PEOU (*β* = 0.231), its theoretical significance implies that ethical concerns act as a distinct psychological barrier (supporting H12). This finding indicates that, in educational contexts, users’ adoption of AIGC tools is not solely driven by functional expectations, but is highly sensitive to ethical implications. Especially in high-risk academic scenarios such as coursework, academic writing, and examinations, users’ awareness of issues like “lack of originality” or “ambiguous responsibility” may directly suppress their intention to use these tools. This result aligns with Shin’s (2021) assertion that “AI-related ethical concerns can directly undermine user trust, thereby influencing decision-making,” and resonates with findings by Zhou et al. (2018) that “AI usage in educational settings is more constrained by moral norms and expectations.

From a path coefficient perspective, the effect of PEC on BI was notably stronger than that of PS, PV, SE, and RE—indicating that users’ risk cognition regarding the likelihood and severity of consequences often requires mediation through PEC to impact behavioral outcomes. In other words, PMT variables serve as upstream cognitive factors influencing PEC, rather than directly predicting behavioral intention. This structural relationship reinforces PEC’s theoretical role as a mediator in ethical cognition and illustrates that educational users are more concerned with whether the technology is “morally appropriate” rather than merely “risky.” A similar structure was confirmed by Jannat et al. (2024), who found that PMT variables influence behavior primarily through ethical anxiety.

In addition, the moderating effect of MS on the PEC → BI relationship was also empirically validated. Specifically, when MS was high, the negative effect of PEC on BI was significantly amplified; when MS was low, the effect was attenuated (supporting H14). This suggests that individuals respond differently to the same ethical issue, depending on their capacity to perceive value conflicts. High-MS individuals tend to internalize ethical concerns more strongly, translating them into avoidance behaviors (Strahovnik, 2018). This result is consistent with Rest et al.’s (1999) moral development theory, which posits moral sensitivity as a prerequisite for moral judgment, and echoes Vance et al.’s (2012) empirical findings that individuals with higher MS are more prone to triggering behavioral defense mechanisms. Notably, compared with general cognitive variables in TAM and PMT, MS represents a relatively stable psychological trait, making its moderating effect more context-independent. In education—where norms, fairness, and responsibility are highly emphasized—such trait-level effects are particularly salient. For instance, teachers or postgraduate students typically exhibit a stronger awareness of academic norms, and thus tend to score higher on MS than undergraduate students. This difference may help explain the observed variation in the PEC → BI inhibitory path across user subgroups. Taken together, PEC not only functions as an outcome of risk perception but also serves as a crucial bridge between moral judgment and behavioral response. Meanwhile, MS operates as a psychological threshold at the individual level—only when users possess sufficient ethical awareness can the moral conflicts represented by PEC translate into behavioral inhibition (Crowell et al., 2008). This finding further consolidates the moderating role of MS within the ethical pathway and adds depth to existing AI ethics adoption models.

Furthermore, our findings regarding the interplay between functional value and ethical concerns resonate with the broader discourse on AI-driven educational transformation. As noted by Naidoo (2023) and Rughiniș et al. (2025), the sustainable integration of AI requires balancing technological advancement with human-centric ethical considerations. Our model provides empirical evidence for this balance by demonstrating that adoption is not a linear function of utility alone. Rather, it is the result of a dynamic trade-off: while PU drives the ‘engine’ of adoption, PEC acts as a critical ‘brake’—particularly for morally sensitive users. Therefore, achieving the sustainable transformation envisioned in recent scholarship depends on establishing a ‘function-ethics equilibrium,’ where efficiency gains are matched by equally robust ethical safeguards RE to foster long-term trust.

### 5.4 Practical implications

The findings of this study offer concrete guidelines for educational AIGC stakeholders. First, for platform designers, the significant impact of RE on lowering PEC suggests that “visible ethical guardrails” are essential. Since users’ trust relies heavily on external safeguards, designers should embed features such as real-time plagiarism checks, clear data usage transparency badges, and one-click citation generation directly into the interface. These design cues can psychologically reassure users that the system is governed by safe protocols, thereby mitigating ethical anxiety.

Second, for educators and policymakers, the moderating role of MS necessitates a differentiated approach to guidance. Our results show that users with high MS are more prone to avoiding AIGC due to ethical fears. For this group, institutions should provide clear “safe-use lists” and definitive integrity policies to alleviate their uncertainty. Conversely, for users with low MS who may not naturally perceive ethical risks, educational programs should focus on “ethical awakening”—using case studies of AI misuse to heighten their sensitivity and prevent reckless adoption.

Third, regarding the link between risk perception and usefulness, developers must prioritize “explainability” to lower cognitive costs. Since high perceived severity reduces utility by forcing users to verify outputs, future tools should provide confidence scores or source attribution. This would reduce the ‘cognitive verification cost,’ thereby enhancing both perceived usefulness and adoption intention.

### 5.5 Limitations and directions for future research

Despite validating a dual-path model of AIGC adoption in education, this study has several limitations. First, the sample pooled university students, teachers, and platform employees. Although our regression analysis indicated no significant impact of occupation on behavioral intention, the sample size of specific subgroups was insufficient to conduct a rigorous Multi-Group Analysis (MGA) in SEM. Different stakeholders may indeed possess distinct ethical concerns. Future research should aim for larger, balanced sample sizes to systematically compare group differences and include broader user groups like K–12 teachers or corporate professionals. Second, the cross-sectional design prevents analysis of behavioral change over time; longitudinal or experimental methods are recommended. Third, the model emphasizes individual cognition, overlooking macro-level influences such as social norms or institutional policies. Lastly, although reliability and validity were confirmed, construct applicability across cultural settings requires further validation. Future studies should explore broader contexts and adopt mixed methods to enrich theoretical and empirical contributions. Furthermore, methodological advancements in generative AI offer new avenues for instrument development. As noted in recent scholarship, Large Language Models (LLMs) show promise in automating the generation and cross-cultural adaptation of psychometric items (Grobelny et al., 2025; Marmolejo-Ramos et al., 2025). Future researchers could leverage these tools to further refine the validity and efficiency of scales used in educational technology acceptance.

## 6 Conclusion

This study developed a dual-pathway model integrating TAM and PMT to examine the adoption of AIGC tools in education, incorporating PEC and MS as key variables. Results from 589 valid responses confirmed that both perceived usefulness and perceived ease of use significantly promote behavioral and continuance intentions. Meanwhile, threat and coping appraisals indirectly shape user behavior via functional and ethical evaluations. PEC exerted a strong negative effect on adoption, an effect significantly moderated by MS—indicating that ethical concerns and individual sensitivity are critical factors in decision-making. Theoretically, this study extends TAM by integrating ethical cognition into sustainable adoption models. Practically, it offers actionable insights for improving AIGC platforms through enhanced usability, risk mitigation, and user-specific ethical strategies. These findings support the development of ethical, inclusive, and resilient educational technologies. Future research should explore longitudinal trends and cultural diversity to further enhance the sustainable and responsible integration of AIGC in education.

## Data Availability

The original contributions presented in the study are included in the article/supplementary material, further inquiries can be directed to the corresponding author.
